# High Prevalence of the EBER Variant EB-8m in Endemic Nasopharyngeal Carcinomas

**DOI:** 10.1371/journal.pone.0121420

**Published:** 2015-03-25

**Authors:** Zhi-chao Shen, Bing Luo, Jian-ning Chen, Yan Chao, Chun-kui Shao, Qian-qian Liu, Yun Wang

**Affiliations:** 1 Department of Medical Microbiology, Qingdao University Medical College, Qingdao, People’s Republic of China; 2 Department of Pathology, The Third Affiliated Hospitals of Sun Yat-sen University, Guangzhou, People’s Republic of China; 3 Department of Clinical Laboratory, Guangdong Provincial Hospital of Traditional Chinese Medicine, Guangzhou, People’s Republic of China; Gustave Roussy, FRANCE

## Abstract

Epstein-Barr virus (EBV)-encoded small RNAs (EBERs) are the most highly expressed transcripts in all EBV-associated tumors and are involved in both lymphoid and epithelioid carcinogenesis. Our previous study on Chinese isolates from non-endemic area of nasopharyngeal carcinoma (NPC) identified new EBER variants (EB-8m and EB-10m) which were less common but relatively more frequent in NPC cases than healthy donors. In the present study, we determined the EBER variants in NPC cases and healthy donors from endemic and non-endemic areas of NPC within China and compared the EBER variants, in relation to the genotypes at BamHI F region (prototype F and f variant), between population groups and between two areas. According to the phylogenetic tree, four EBER variants (EB-6m, EB-8m, EB-10m and B95-8) were identified. EB-6m was dominant in all population groups except for endemic NPC group, in which EB-8m was dominant. EB-8m was more common in endemic NPC cases (82.0%, 41/50) than non-endemic NPC cases (33.7%, 32/95) (*p*<0.0001), and it was also more frequent in healthy donors from endemic area (32.4%, 24/74) than healthy donors from non-endemic area (1.1%, 1/92) (*p*<0.0001). More importantly, the EB-8m was more prevalent in NPC cases than healthy donors in both areas (*p*<0.0001). The f variant, which has been suggested to associate with endemic NPC, demonstrated preferential linkage with EB-8m in endemic isolates, however, the EB-8m variant seemed to be more specific to NPC isolates than f variant. These results reveal high prevalence of EBER EB-8m variant in endemic NPC cases, suggesting an association between NPC development and EBV isolates carrying EB-8m variant. Our finding identified a small healthy population group that shares the same viral strain which predominates in NPC cases. It could be interesting to carry extensive cohort studies following these individuals to evaluate the risk to develop NPC.

## Introduction

Epstein-Barr virus (EBV) is a lymphotropic virus that infects more than 90% of the world's population and is associated with the development of both lymphoid and epithelial tumors, such as Burkitt's lymphoma (BL), Hodgkin's disease (HD), B- and T-cell lymphomas, nasopharyngeal carcinoma (NPC) and gastric carcinoma [1]. Although the virus homogeneously targets populations from all geographic areas, the incidence of the diseases it causes varies drastically. It is well known NPC occurs with a remarkable geographic pattern and is endemic in southern China, Hong Kong, Taiwan and Southeast Asia [2, 3]. What causes these variations in disease incidence is unclear. Multiple factors, including genetic susceptibility, environmental factors and EBV infection are believed to account for the geographical distribution of NPC [4]. Due to the oncogenic potency and the ubiquitous presence in NPC of virus, EBV infection is considered to be an important contributing factor of NPC [5]. Attention has been focused on whether particular EBV strains are prevalent in endemic area and contribute to the development of NPC. Most previous studies characterized the EBV isolates based on restricted fragment length polymorphism (RFLP) at the BamHI F fragment and BamHI W1/I1boudary region, as well as using the strain-specific markers in the EBV nuclear antigen (EBNA) 2 and 3, EBNA1, latent membrane protein (LMP) and BZLF1 loci [6]. The f variant carrying an extra BamHI site in the BamHI F fragment was found more frequent in endemic NPC cases than controls [7, 8]. The del-LMP1 (30-bp deletion) have been clinically associated with tumor aggressiveness and functionally linked to enhanced oncogenic potential [9–11]. Association of LMP1 deletion variant Asp335 with NPC in Hong Kong was reported [12]. Specific EBNA1 or LMP1 subtype ((V-val or China 1) also showed preferential occurrence in NPC biopsies [13, 14]. These observations support the notion of pathogenic strains with NPC. However, some studies found strain differences between geographical locations in various genes such as BARF1, LMP1 and EBNA1 but similar spectra of EBV subtypes among tumors and corresponding healthy populations, suggesting a geographically restricted polymorphism [15–17]. There are epidemiological and functional data indicating that V-val, del-LMP1 or China 1 represents geographically restricted rather than tumor-specific variant [16–22]. Therefore, the geographical distribution of EBV variants and their precise association to diseases remain unresolved.

EBV-encoded small RNAs (EBERs, EBER1 and EBER2) are the most abundant viral transcripts expressed in all EBV-associated tumors. Recently, EBERs have been reported to play important roles in both lymphoid and epithelioid carcinogenesis, such as BL [23, 24], B-cell lymphoma [25, 26], gastric carcinoma [27] and NPC [28, 29]. EBER1 and EBER2 are 167 and 172 nucleotides long, respectively, and separated by a 161-bp non-coding spacer region at prototype B95-8 genome of EBV. Early sequence analysis on EBV strains (B95-8, AG876 and P3HR-1) has identified two patterns of EBER variations, which correspond to B95-8 and AG876/P3HR-1, respectively, and found that most of the type-specific changes occur at the161-bp non-coding spacer region [30]. The polymorphism of EBER genes has not received much attention since it has been generally believed that the coding sequences of EBER1 and EBER2 are conserved among various EBV isolates [30, 31]. Only a few studies used EBER locus to distinguish EBV isolates, and found that P3HR-1 type EBER was predominant and B95-8 type was less common in isolates from different EBV-related individuals in America, Germany, Italy and South Africa [31–35]. Interestingly, in our previous study on northern Chinese isolates from NPC non-endemic area, three major variants of EBER genes, designated as EB-6m, EB-8m and EB-10m, were identified. EB-6m, which was identical to P3HR-1 EBER, was dominant in each population group, while the new variants, EB-8m and EB-10m, which shared six common mutations at EBER2 coding region, showed relatively higher frequency in NPC cases (13/47, 27.7%) than in EBV-associated gastric carcinoma (EBVaGC) cases (1/50, 2.0%) and healthy donors (0/57) [36]. In endemic area of NPC in southern China, only one report explored the EBER variations in a small size of NPC samples and found all the assayed NPC samples (20/20) had a similar sequence with EB-8m [37]. These results suggest specific EBER variants may have geographical distribution and associate with NPC. However, more evidence, especially that from NPC endemic areas, is needed to support the view.

Therefore, in the present study, we determined the EBER variants in NPC cases and healthy donors from endemic and non-endemic areas of NPC within China, and compared the EBER variants, in relation to polymorphism at the BamHI F fragment, between two areas and between NPC cases and healthy donors to verify a NPC-associated variation of EBER.

## Materials and Methods

### Ethics statement

This study was approved by the Medical Ethics Committee of Qingdao University Medical College. Written informed consents were taken from all the patients and healthy donors and ethical guidelines under Declaration of Helsinki were followed.

### Subjects

In NPC endemic area, 21 cases of fresh and 41 cases of paraffin-embedded NPC tissues were collected from the First and Third Affiliated Hospitals of Sun Yat-sen University, Guangzhou, southern China, and 161 throat washing (TW) samples of healthy donors, were collected from the Guangdong Provincial Hospital of Traditional Chinese Medicine, Guangzhou, southern China. In NPC non-endemic area, 224 cases of EBV-positive samples, including 120 NPC tissues and 104 TW samples from healthy donors, which were identified in our previous study [38], were included in the present study. The NPC samples were collected from the major hospitals in Shandong Province, northern China, and the healthy controls were from the same geographic regions. Patients and healthy donors were interviewed to verify whether their ancestral home was within the endemic or non-endemic area.

### Preparation of DNA

Genomic DNA from the fresh tissues and TW samples was carried out using the standard method with proteinase K digestion and phenol chloroform purification, and the genomic DNA from the paraffin-embedded NPC tissue was prepared according to the manufacturer’s instructions (QIAamp DNA FFPE Tissue Kit, QIAGEN GmbH, Hilden, Germany).

### Sequencing of EBER genes

Nested-polymerase chain reaction (PCR) and direct sequencing were used to detect the sequences of EBER genes as described in our previous study [36]. The sequencing results were checked by Chromas software to find out whether there were heterozygous sequences. Nucleotide sequences of EBERs across nucleotides 6629 to 7128 of B95-8 prototype (GenBank accession number V01555) [39] were compiled from our samples and were compared with the B95-8 reference genome as well as the published sequences. Alignments between sequences were analyzed using the Clustal W method of DNAStar software (DNASTAR, Inc, version 7.0).

### Phylogenetic analysis

Phylogenetic analysis was performed using the Molecular Evolutionary Genetics Analysis (MEGA) software, version 5.0 [40], by Neighbor-joining algorithm, based on multiple sequence alignments of the nucleotide sequences of EBER genes. Bootstrap analysis of 1000 replicates was performed on the tree to determine the confidence.

### EBV genotype analysis at BamHI F region

PCR and RFLP were employed for distinguishing prototype F and f variant as described previously [41]. To confirm the specificity of PCR and RFLP reactions, PCR products of the representative samples were purified and directly sequenced in both directions, and the data were analyzed with Chromas software and DNAStar software, respectively.

### Statistical analysis

The differences in distribution of EBER variants, prototype F and f variant, the combinational genotypes at two gene loci and the relationship between EBER and BamHI F polymorphisms were compared by the two sided Fisher’s exact test, and the differences in frequency of the specific variant between NPC cases and healthy donors as well as between NPC endemic and non-endemic areas were assayed by Chi-square test. McNemar’s test was used to compare the frequency of the specific variant of EBER with that of f variant in endemic NPC isolates which were available of sequences in two gene loci. Differences were considered significant when *p* values less than 0.05.

## Results

### Sequence variations of EBER genes and determination of EBER variants

**Fig 1:**
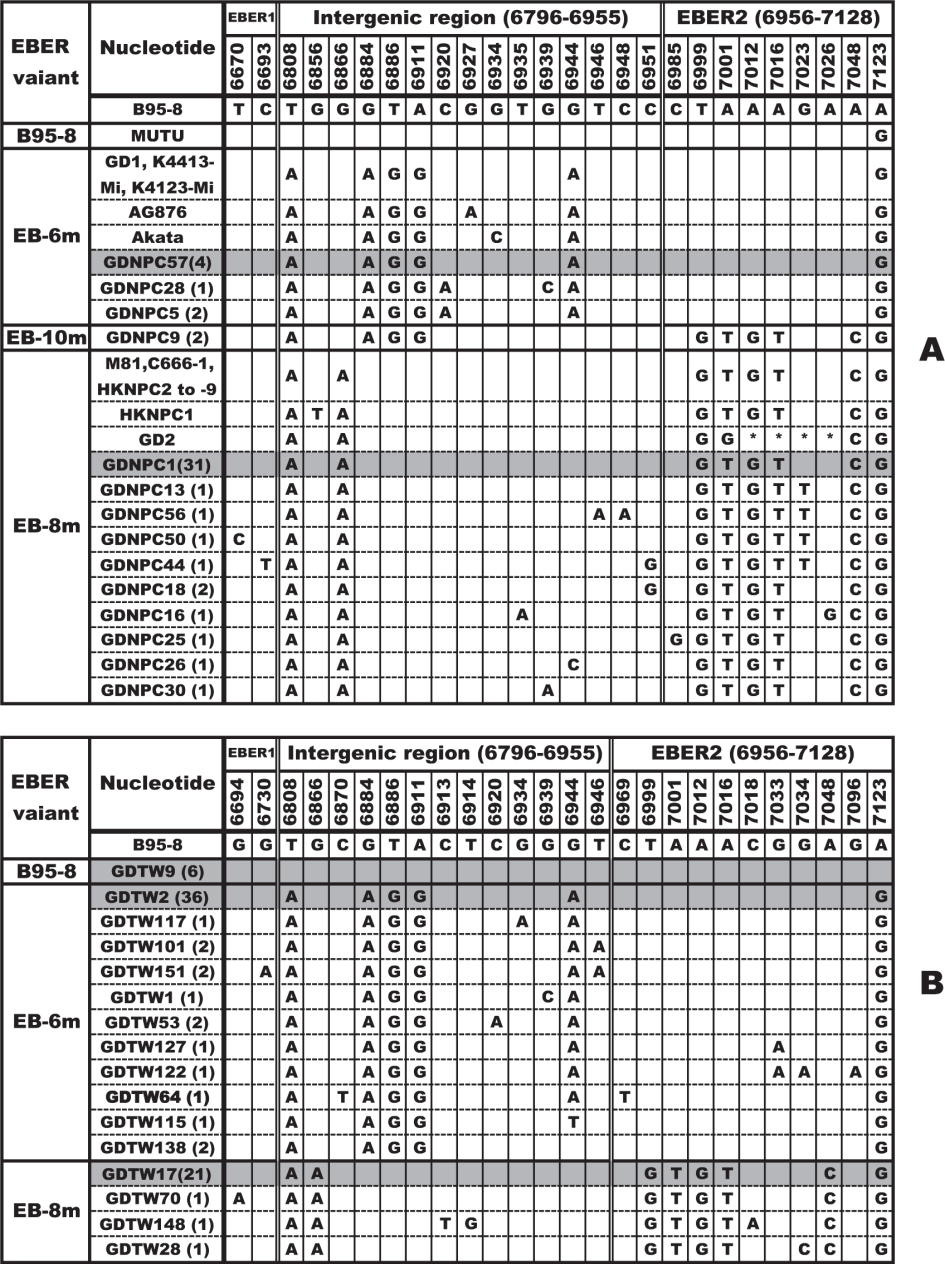
EBER variations in NPC cases (A) and healthy donors (B) from NPC endemic area in China. Numbers in the second top correspond to the nucleotide positions under which the B95-8 prototype nucleotide sequence is listed. Only nucleotides different from B95-8 are indicated. An asterisk indicates a deletion of a nucleotide. The changes in coding regions of EBER1 and EBER2 and non-coding region between EBER1 and EBER2 are separated by double lines and the positions are shown in the top. Different variants are noted to the left column, while the isolates showing identical sequences in each population group are listed by a representative isolate in the second column. The followed numbers in the parentheses denote the amount of the identical sequences from the same population group. The consensus sequence of each variant is shaded. The EBER sequences of 19 sequenced EBV genomes (B95-8, K4413-Mi, K4123-Mi, AG876, MUTU, Akata, GD1, GD2, M81, HKNPC1, C666-1 and HKNPC2 to −9) were taken from GenBank (V01555, KC440852, KC440851, DQ279927, KC207814, KC207813, AY961628, HQ020558, KF373730, JQ009376, KJ411974 and KF992564 to KF992571) [39, 42–49].

**Fig 2:**
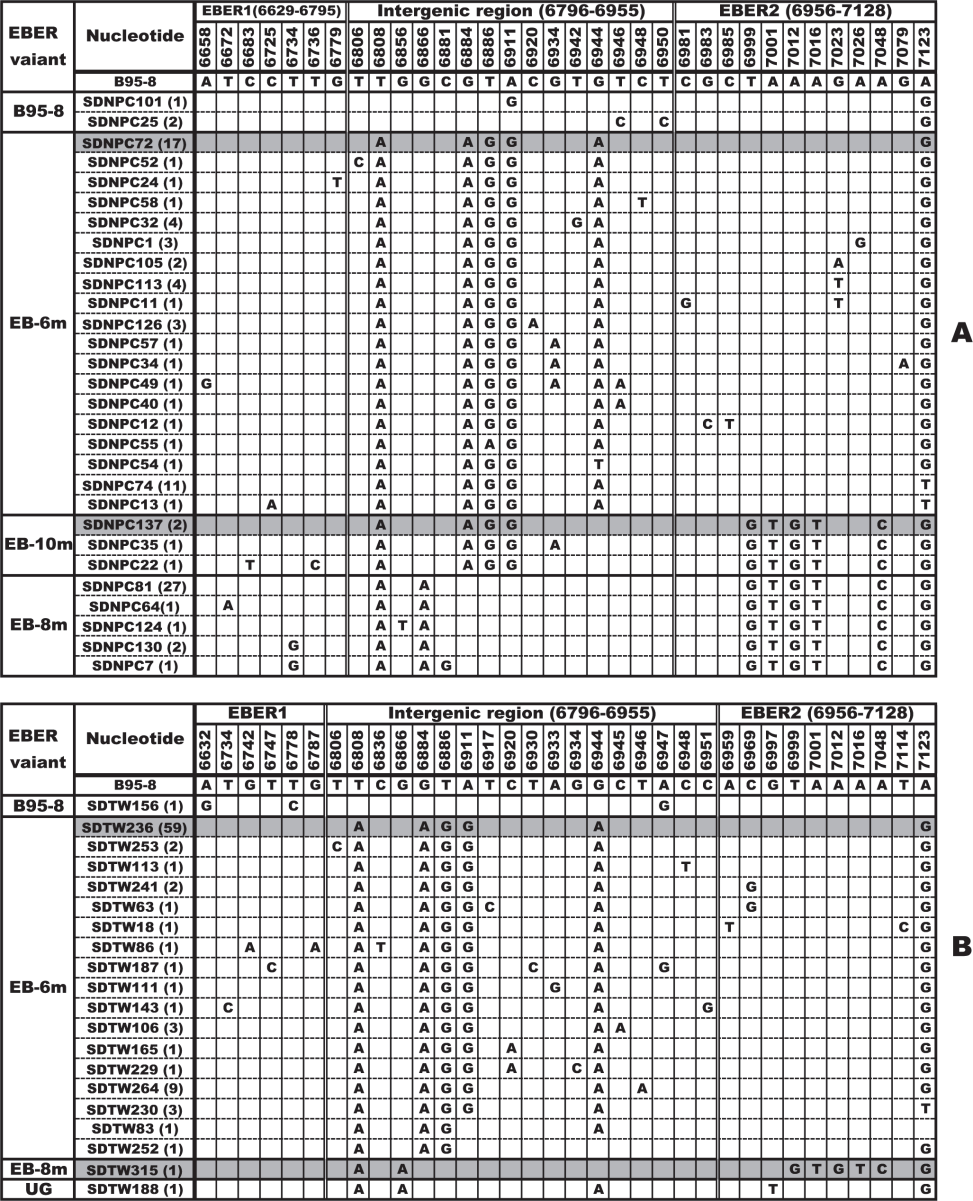
EBER variations in NPC cases (A) and healthy donors (B) from NPC non-endemic area in China. Numbers in the second top correspond to the nucleotide positions under which the B95-8 prototype nucleotide sequence is listed. Only nucleotides different from B95-8 are indicated. The changes in coding regions of EBER1 and EBER2 and non-coding region between EBER1 and EBER2 are separated by double lines and the positions are shown in the top. Different variants are noted to the left column, while the isolates showing identical sequences in each population group are listed by a representative isolate in the second column. The followed numbers in the parentheses denote the amount of the identical sequences from the same population group. The consensus sequence of each variant is shaded.

For cases in endemic area of NPC, EBER fragment was successfully amplified and sequenced in 124 samples, including 50 NPC biopsies and 74 TW samples of healthy donors. The sequencing results showed the presence of a single EBER sequence in 50 NPC and 68 TW samples, whereas the remaining 6 TW samples displayed dual EBER sequences. In total, 130 isolates were obtained from 124 samples (Fig. 1). For cases in non-endemic area of NPC, we have determined the variations of EBER in 47 NPC biopsies and 57 TW samples of healthy donors in the previous study [36]. In the present study, another 48 NPC biopsies and 35 EBV-positive TW samples from healthy donors in the same populations were further assayed to determine the EBER variants, and they showed similar variation patterns with previous cases. The sequence variations of EBER in all the 95 NPC cases and 92 healthy donors are illustrated in Fig. 2.

The EBER variants were determined based on the common signature mutations across nucleotides 6629 to 7128, proposed in our previous study [36]. Four variants, designated as EB-6m, EB-8m, EB-10m and B95-8, were identified. Relative to B95-8 sequence, the consensus sequence of EB-6m exhibited five common mutations at 161-bp space region and one at EBER2 coding region, while that of EB-8m carried six common mutations at EBER2 coding region and two at 161-bp space region. The consensus sequence of EB-10m had the first four nucleotide substitutions of EB-6m and the last six substitutions of EB-8m, and that of B95-8 variant was identical to B95-8 prototype (Figs. 1 and 2). Dual infection had double signals at the same signature nucleotide positions. As shown in Figs. 1 and 2, most of the isolates in each variant group only harbored the signature point mutations of each variant, while a minority of isolates in each group showed additional mutations at other positions and some isolates had different changes at one or two of the common mutations. All the 317 EBER nucleotide sequences were submitted to the Genbank database with accession numbers KP195385 to KP195701.

The EBER sequences of 19 EBV genomes reported to date are also shown in Fig. 1A. B95-8 (V01555), derived from a North American case of infectious mononucleosis [39], was the first completely sequenced EBV genome. Most recently, two more EBV genomes, K4413-Mi (KC440852) and K4123-Mi (KC440851), in immortalized human B lymphocyte cell lines which were established from healthy blood donors in America, were sequenced [42]. AG876 (DQ279927), MUTU (KC207814) and Akata (KC207813) were originated from BL patients in Western Africa, Eastern Africa and Japan, respectively [43, 44]. The EBV genomes isolated from NPC patients in southern China include GD1 (AY961628) [45], GD2 (HQ020558) [46], M81 (KF373730) [47], HKNPC1 (JQ009376) [48, 49], C666-1 (KJ411974) and HKNPC2 to-9 (KF992564 to KF992571) [49]. The EBER sequence of MUTU was identical to that of B95-8 except for the presence of mutation at position 7123, which was arranged in B95-8 group. GD1, K4413-Mi, K4123-Mi, AG876 and Akata belonged to EB-6m group, and other 12 genomes belonged to EB-8m group. Three genomes (AG876, Akata and HKNPC1) had an additional mutation except the common mutations. GD2 had a 23-bp deletion (B95-8 coordinates 7007–7029: GTGCTACCGACCCGAGGTCAAGT) and a different change at one of the eight common mutations of EB-8m at position 7001 (Fig. 1A).

### Phylogenetic analysis of the EBER nucleotide sequences

**Fig 3:**
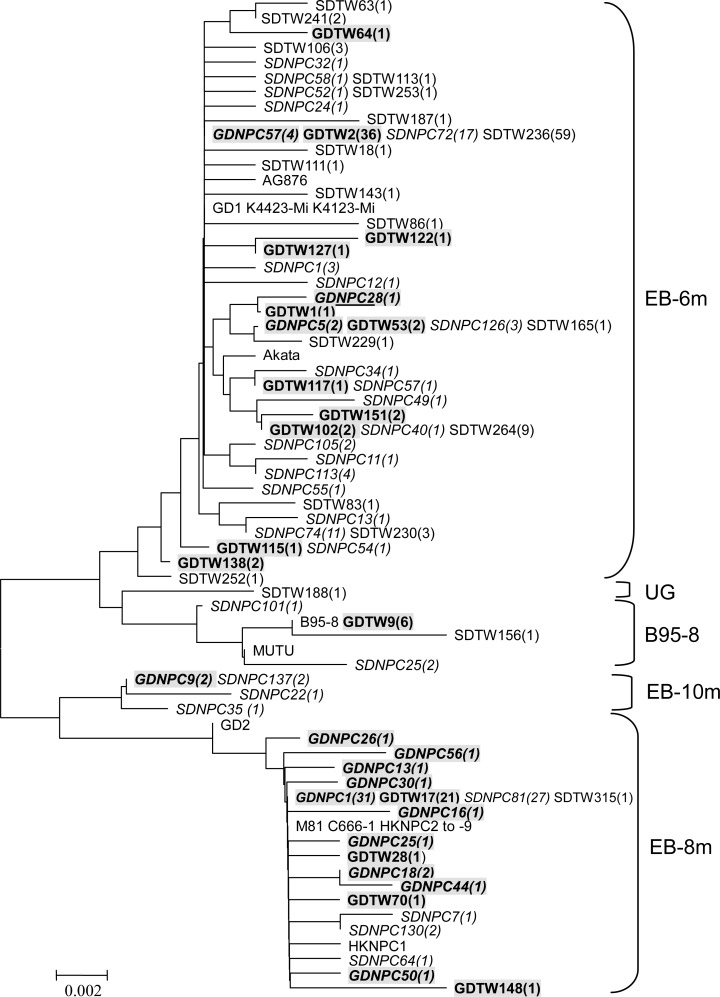
Phylogenetic tree based on sequences of EBER genes by neighbor-joining method. All the 317 determined EBER sequences in our study and the 19 EBER sequences of reported EBV genomes ((B95-8, K4413-Mi, K4123-Mi, AG876, MUTU, Akata, GD1, GD2, M81, HKNPC1, C666-1 and HKNPC2 to-9) [39, 42–49] are included in the phylogenetic tree. Isolates from endemic area are black shaded and the isolates from NPC cases are shown in italic. Population group: GDNPC and GDTW, NPC cases and healthy donors from Guangzhou, Guangdong Province, the endemic area of NPC in China; SDNPC and SDTW, NPC cases and healthy donors from Shandong Province, the non-endemic area of NPC in China. The isolates showing identical sequences in each population group are listed by a representative isolate, and the followed numbers in the parentheses denote the amount of the identical sequences from the same population group. The scale shown in the lower left shows the evolutionary distance.

To strengthen the classification of the above patterns as true strains, all the 317 determined EBER sequences in our present study and previous study [36] and the 19 EBER sequences of reported EBV genomes [39, 42–49] were used to construct a phylogenetic tree. According to the resulting phylogenetic tree (Fig. 3), these isolates were segregated in four lineages, just the same as the four variant groups shown in Figs. 1 and 2. These similar groupings confirmed the classification based on the specific mutations. Except for the four major branches, one isolates (SDTW188) derived from healthy donors in NPC non-endemic area was located in a separate branch and was classified in “Ungrouped (UG)”group as it showed different variation pattern and could not be assigned to any of the four groups (Figs. 2B and 3). The tree also revealed the phylogenetic relationships among various EBV isolates. The isolates with EB-10m sequence seemed to be recombinant strains of the two major lineages, EB-6m and EB-8m. They were positioned near to EB-8m isolates due to the presence of more mutations identical to EB-8m. The B95-8 sequence was closer to EB-6m than EB-8m (Fig. 3).

### Distribution of EBER variants in NPC cases and healthy donors

The frequencies of EBER variants in NPC cases and healthy donors in both areas are summarized in Table 1. In NPC endemic area, EB-8m and EB-6m were detected in most samples. The distribution of EBER variants in NPC cases was significantly different from that in healthy donors (*p*<0.0001). The EB-8m variant was found in 41/50 (82.0%) NPC cases, whereas in 24/74 (32.4%) healthy donors. This difference was significantly different (*p*<0.0001). In NPC non-endemic area, the EB-6m was the most predominant variant in both population groups, followed by EB-8m. The distribution of EBER variants in NPC cases was also significantly different from that in healthy donors (*p*<0.0001) with higher frequency of EB-8m in NPC cases (33.7%, 32/95) than in healthy donors (1.1%, 1/92) (*p*<0.0001).

**Table 1:** Distribution of EBER and BamHI F variants in NPC cases and healthy donors in endemic and non-endemic areas of NPC in China.

Variant		Endemic area		Non-endemic area	
		NPC No. (%)	Healthy donors No. (%)	NPC No. (%)	Healthy donors No. (%)
EBER					
	EB-6m	7 (14.0%)	**44 (59.5%)**	**56 (58.9%)**	**89 (96.7%)**
	EB-8m	**41 (82.0%)**	**22 (29.7%)**	**32 (33.7%)**	1 (1.1%)
	EB-10m	2 (4.0%)	0	4 (4.2%)	0
	B95-8	0	2 (2.7%)	3 (3.2%)	1 (1.1%)
	EB-6m+B95-8	0	4 (5.4%)	0	0
	EB-6m+EB-8m	0	2 (2.7%)	0	0
	UG	0	0	0	1 (1.1%)
	Total	50 (100%)	74 (100%)	95 (100%)	92 (100%)
BamHI F					
	F	**16 (29.1%)**	**43 (60.6%)**	**105 (87.5%)**	**98 (94.2%)**
	f	**38 (69.1%)**	**24 (33.8%)**	15 (12.5%)	6 (5.8%)
	F+f	1 (1.8%)	4 (5.6%)	0	0
	Total	55 (100%)	71 (100%)	120 (100%)	104 (100%)

Numbers in bold indicate the major strain(s) in each population.

The distributions of EBER variants between endemic and non-endemic NPC cases, and between healthy donor groups from NPC endemic and non-endemic areas were significantly different (NPC, *p*<0.0001; healthy donors, *p*<0.0001). EB-8m was more common in endemic NPC cases (82.0%, 41/50) than in non-endemic NPC cases (33.7%, 32/95) (*p*<0.0001), and it also demonstrated higher frequency in healthy donors from NPC endemic area (32.4%, 24/74) than in healthy donors from NPC non-endemic area (1.1%, 1/92) (*p*<0.0001) (Table 1).

### BamHI F polymorphism in NPC cases and healthy donors

For cases in endemic area of NPC, the BamHI F region was successfully amplified in 126 samples, including 55 NPC biopsies and 71 TW samples of healthy donors. For cases in non-endemic area of NPC, the genotypes at BamHI F region were available in 224 samples, including 120 NPC biopsies and 68 TW samples of healthy donors which were determined in our previous study [38] and 36 TW samples of healthy donors which were assayed in the present study. In NPC endemic area, the f variant was the dominant type in NPC (70.9%, 39/55), but not in healthy donors (39.4%, 28/71). This difference was statistically different (*p* = 0.0004). In NPC non-endemic area, the dominant type was prototype F in both NPC (87.5%, 105/120) and healthy donors (94.2%, 98/104), and the distribution of prototype F and f variant in NPC cases and healthy donors did not differ statistically (*p* = 0.085) (Table 1). Similar to EB-8m variant, the f variant was frequently detected in endemic NPC (70.9%, 39/55) than in non-endemic NPC cases (12.5%, 15/120) (*p*<0.0001), and it showed higher frequency in healthy donors from NPC endemic area (39.4%, 28/71) than in healthy donors from NPC non-endemic area (5.8%, 6/104) (*p*<0.0001) (Table 1).

### Association of EBER variants with BamHI F genotypes

By integrating the EBER variants with BamHI F genotypes, 9 independent combinational genotypes (BamHI F/EBER) were identified in 292 non-coinfection samples which were available of sequences in two gene loci (Table 2). Similar to EB-8m, the distribution of the combinational genotypes were significantly different between NPC cases and healthy donors (p<0.0001) in both areas as well as between endemic and non-endemic areas (*p*<0.0001). The F/EB-6m was the most common genotype in each population group except for endemic NPC group, in which the f/EB-8m was dominant. The f/EB-8m was only detected in endemic cases with a higher frequency in NPC (64.0%, 32/50) than in healthy donors (21.0%, 13/62) (*p*<0.0001), whereas the F/EB-8m was detected in both endemic and non-endemic areas with a higher frequency in NPC cases (35.2%, 31/88) than in healthy donors (1.1%, 1/92) in non-endemic area (*p*<0.0001), and also with a relatively high frequency in endemic NPC cases (18.0%, 9/50) than corresponding healthy donors (11.3%, 7/62). Of the 13 reported NPC strains in NPC endemic area, 9 (69.2%) (GD2, C666-1, M81, HKNPC1, HKNPC3, HKNPC4, HKNPC5, HKNPC8 and HKNPC9) contained f/EB-8m variant, 3 (23.1%) (HKNPC2, HKNPC6 and HKNPC7) belonged to F/EB-8m variant and 1 (7.8%) (GD1) belonged to F/6m variant. The distribution of EBER variants, BamHI F genotypes or the combinational genotypes of them in 13 reported NPC strains was similar to that in endemic NPC isolates in the present study (*p* = 0.592, 0.790, 0.879, respectively) (Tables 1 and 2). Of the other six strains, K4413-Mi, K4123-Mi, AG876 and Akata belonged to F/EB-6m variant, and B95-8 and MUTU belonged to F/B95-8 variant.

**Table 2:** Comprehensive analysis of EBV genotypes at BamHI F and EBER loci in NPC cases and healthy donors from endemic and non-endemic areas of NPC in China.

Genotype		Endemic area		Non-endemic area	
BamHI F	EBER	NPC No. (%)	Healthy donors No. (%)	NPC No. (%)	Healthy donors No. (%)
f	EB-6m	0	7 (11.3%)	10 (11.4%)	6 (6.5%)
f	EB-8m	**32 (64.0%)**	**13 (21.0%)**	0	0
f	EB-10m	2 (4.0%)	0	1 (1.1%)	0
f	B95-8	0	2 (3.2%)	0	0
F	EB-6m	7 (14.0%)	**33 (53.2%)**	**40 (45.5%)**	**83 (90.2%)**
F	EB-8m	**9 (18.0%)**	7 (11.3%)	**31 (35.2%)**	1 (1.1%)
F	EB-10m	0	0	3 (3.4%)	0
F	B95-8	0	0	3 (3.4%)	1 (1.1%)
F	UG	0	0	0	1 (1.1%)
Total		50 (100%)	62 (100%)	88 (100%)	92 (100%)

Numbers in bold indicate the major strain(s) in each population.

We noted that the distribution of EBER variants in cases with f variant EBV was different from that in cases with prototype F EBV in NPC endemic area (*p*<0.0001). Forty-five of 56 (80.4%) cases with f variant EBV had EB-8m variant, whereas 40 of 56 (71.4%) cases with prototype F EBV had the EB-6m variant. Due to the small number of f variant 1isolates in non-endemic area, the difference of the distribution of EBER variants between f variant EBV and F variant EBV was not analyzed (Table 2). We also compared the frequency of EB-8m with that of f variant in 63 endemic NPC isolates (50 cases of the present study and 13 reported NPC strains) which were available of sequences in two gene loci by McNemar’s test, and found that the frequency of EB-8m was higher than that of f variant (*p* = 0.016, odds ratio: 6.000, 95% confidence interval: 1.393–25.836) (Table 3).

**Table 3:** Comparison of the frequency of EB-8m with that of f variant in endemic NPC isolates.

	f variant	non-f variant	Total	*p* ^†^	OR^‡^	95% CI^§^
EB-8m	41	12	53	0.016	6.000	1.393–25.836
non-EB-8m	2	8	10			
Total	43	20	63*			

*The 63 isolates include 50 NPC cases of the present study and 13 reported NPC strains [45–49].

^†^ *p*-value was obtained from McNemar’s test.

^‡^OR: odds ratio.

^§^95% CI: 95% confidence interval.

## Discussion

This study represents the first comparison of EBER variations in isolates from the NPC endemic area of southern China with those from the NPC non-endemic area of northern China. Phylogenetic analysis identified four distinctive EBER variants (EB-6m, EB-8m, EB-10m and B95-8) in Chinese isolates. The EB-6m was dominant in healthy donors from both areas and non-endemic NPC cases, but not in endemic NPC cases, in which EB-8m was dominant. In NPC endemic area, EB-8m demonstrated very higher frequency in NPC cases (82.0%, 41/50) than in healthy donors (32.4%, 24/74) (*p*<0.0001). In NPC non-endemic area, although EB-8m was not the dominant variant, it also demonstrated higher frequency in NPC cases (33.7%, 32/95) than in healthy donors (1.1%, 1/92) (*p*<0.0001). It is obvious that EB-8m was more common in endemic NPC cases than in non-endemic NPC cases (*p*<0.0001). In addition, although in a small subset of cases (17 cases), our previous study showed that most of NPC cases harbor the same EBV subtype in tumor biopsies and matched TW samples defined by multiple loci, including EBER, EBNA1, EBNA3C, and RFLP at the BamHI F fragment and BamHI W1/I1boudary region [50]. This is consistent with the literature showing that identical genotype of EBV existing in matched TW sample and tumor tissue of a single NPC patient [51, 52], suggesting that the EBV strain in tumor biopsies and TWs might be derived from a similar origin. Taken together, these results indicate a close association of EBV isolates with EB-8m variant and NPC, especially endemic NPC. The phylogenetic tree based EBER genes also supports this view because most of the endemic NPC isolates and all the 12 reported EBV strains (GD2, C666-1, M81, HKNPC1 to −9) established from NPC tissues in southern China [46–49] were clustered in EB-8m branch, while most of the isolates from healthy donors in both areas and isolates from non-endemic NPC cases were clustered in EB-6m branch, along with another NPC strain (GD1) established from saliva of a NPC patient in southern China [45] and four of the six strains (K4413-Mi, K4123-Mi, AG876 and Akata) from non-NPC individuals in NPC non-endemic areas, including America, Western Africa and Japan [42–44].

The frequent association of the f variant, V-val subtype, del-LMP1 or China 1 subtype with endemic NPC was also reported in previous studies [7, 8, 12–14, 51, 53, 55, 56]. However, the distribution of EBER-8m in Chinese isolates has some differences with that of V-val subtype, del-LMP1 and China 1 subtype. First, the V-val, del-LMP1 and China 1 were also dominant in healthy/non-malignant donors and non-NPC tumors (72.0%, 78.1% and 72.5% respectively) [13, 14, 19, 20, 41, 53–56] in endemic area although they were highly prevalent in NPC cases (100%, 91.5% and 89.5% respectively) [12–14, 20, 51, 53, 55–57]. Second, the V-val, del-LMP1 and China 1 were also common in isolates from northern China (78.0%, 76.5% and 64.4% respectively) [13, 17, 18, 20, 21, 57, 58]. Different to V-val, del-LMP1 and China 1, previous studies showed that the f variant was observed in most of the endemic NPC cases (85.7%, 24/28 and 67.3%, 33/49 respectively in two separate studies) [7, 8] but fewer in healthy donors (7.7%, 1/13 and 17.6%, 3/17 respectively in two separate studies) [7, 54] and non-NPC tumor cases (1/19 T cell lymphoma and 9/53 EBVaGC) from endemic area [41, 54, 59]. In NPC non-endemic areas, such as northern China, Japan, Korea, Europe, North America, South America and Africa, the f variant was rarely observed ignoring the source [6, 21, 38]. Thus, the f variant seems only prevalent in endemic NPC cases. The present study examined a larger sample size of NPC and healthy donors from endemic and non-endemic areas in China and further confirms this distribution.

In early studies, P3HR-1 type EBER (EB-6m variant) and B95-8 type EBER (B95-8 variant) have been identified and EB-6m was the most predominant variant in isolates from different EBV-related individuals in different geographical regions, including 13/16 HD cases [31] and 19/24 posttransplantation lymphoproliferative disorders in America [32], 31/41 malignant and benign disorders in Germany [33], 14/15 human immunodeficiency virus-1-seropositive individuals and 7/11 SCID mouse tumors induced by inoculation of EBV-positive peripheral blood mononuclear cells from 11 healthy human donors in Italy [34], 18/21 NPC tissues in South Africa [35], 6/8 lymphoblastoid cell lines derived from cancer patients in Korea [60] and 48/50 EBVaGC tissues in northern China [36]. EB-8m and EB-10m were not detected in isolates listed above except for one isolate in EBVaGC from northern China and 2 lymphoblastoid cell lines derived from cancer patients in Korea, which carried EB-8m variant [36, 60]. Zhang et al. reported a similar sequence with EB-8m in 20/20 of NPC and 4/4 of normal nasopharynx tissues in Guangzhou [37]. Although the data about EBER variations are relatively sparse by now, our and other researcher’s data suggest the significant disease association of EBER variants in addition to the geographical distribution factor. Similar to prototype F and f variant, the EB-6m variant appears to be prevalent in wild isolates from non-endemic area of NPC ignoring source (NPC and non-NPC) as well as in isolates from healthy donors in NPC endemic area in southern China, while the EB-8m variant appears to occur preferentially in endemic NPC cases. Further investigation in more geographical locations is needed to confirm this suggestion.

Interestingly, a preferential linkage between BamHI F genotypes and EBER variants was found in NPC endemic area. The f variant isolates tended to be mainly correlated with the EB-8m variant, and the prototype F isolates tended to be mainly correlated with the EB-6m variant. The correspondence of f variant to EB-8m was especially prominent in endemic NPC cases since all the 9 f variant EBV genomes derived from NPC and 32/34 f variant isolates in the present study carried EB-8m and 2/34 carried its related variant, EB-10m. In previous studies, a linkage between EBNA3C types (type 1 and 2) with EBNA1 subtypes (V-val and P-thr) or LMP1 variants (China 1 and China 2) was demonstrated in northern Chinese isolates and Hong Kong NPC isolates, respectively [12, 18]. The investigation of multiple polymorphic sites in the EBV genome might be beneficial for making reliable assessments related to EBV-associated diseases. As shown in Table 2, either the f/EBER-8m or the F/EBER-8m was preferentially found in NPC cases than in healthy donors. We also found a preferential linkage between EB-8m variant and del-LMP1 and a preference of EB-8m/del-LMP1 in NPC cases over healthy donors (data not shown). The findings further indicate the close relation of EB-8m with NPC.

Although the f variant demonstrated similar distribution and preferential linkage with EB-8m in endemic isolates, the EB-8m variant seems to be more specific to NPC isolates than f variant since the frequency of EB-8m variant in NPC isolates was higher than f variant (*p* = 0.016, odds ratio: 6.000) (Table 3). Due to the relatively small size of the NPC cases in the present study, we could not reach a clear-cut conclusion about this issue by now. It needs further study on large sizes of the NPC samples. The phylogenetic tree based on whole EBV genomes of the 19 reported genomes showed that the 12 EBV genomes which were isolated from endemic NPC tumors were clustered in one major branch and segregated from other 7 genomes [47, 49]. Coincidentally, all the 12 genomes carried EB-8m variant, whereas other 7 genomes carried EB-6m variant or B95-8 variant. The reported NPC strains derived from southern China demonstrated similar distribution of EBER variants with the NPC isolates in the present study. Thus, the EB-8m variant may serve as a marker of the representative EBV strains prevalent in endemic NPC.

Comparisons between NPC endemic and non-endemic areas in China showed that the EB-8m or f variant was also relatively more frequent in healthy donors from endemic area than healthy donors from non-endemic area. These may raise the possibility that the occurrence of EB-8m or f variant in endemic NPC is due to a geographically based polymorphism. However, the preference of EB-8m or f variant in endemic NPC cannot be only contributed to the geographical distribution because either EB-8m or f variant was less common than EB-6m or prototype F in healthy donors but they were dominant in NPC cases. Furthermore, the EB-8m variant was also more frequent in NPC cases than in healthy donors in non-endemic area of NPC in China. The sequence variations of EBER in NPC were similar to those in healthy donors, suggesting that the EBV strains in NPC originate from the viral strains pre-existing within the background population. Therefore, the selectively predominance of EB-8m variant in NPC cases indicates that EBV isolates with EB-8m variant may be high-risk and there may be a selective tropism for these isolates during the development of NPC. The low prevalence of the so-called high-risk EBV isolates in healthy individuals may be attributed to their rare presence, whilst distinctive properties of the isolates such as fatal prognosis and/or host-cell preference may have relationships with disease profiles that result in the accumulation of the isolates in the patient groups. Full scale epidemiological studies and functional analysis of this specific EBV strain are required to confirm this hypothesis. The epidemiological and functional studies suggest that host genetic susceptibility plays crucial roles in mediating EBV infection, maintenance of latent infection and cell transformation [4]. The preference of EBV isolates with EBER-8m variant in endemic NPC biopsies might also be based on inheritable or acquired host susceptibility. Our finding identified a small healthy population group that share the same viral strain which predominate in NPC cases. It could be interesting to carry extensive cohort studies following these individuals longitudinally to evaluate their genetic susceptibility and the risk to develop NPC.

The role of EBER1 and EBER2 in oncogenesis has been demonstrated recently [23–29].They contribute to oncogenesis by promoting growth of tumor cells and modulating innate immunity in patients with NPC and BL [23, 24, 28, 29, 61]. As mentioned in our previous study, some of the six common changes of EB-8m (nucleotide positions in EBER2 transcript: 44, 46, 57, 61, 93 and 167) identified in the EBER2 are located in the stem-loops, such as nucleotides at 44, 46, 57 and 61 [36]. The stem-loops are consisted in the secondary structures of EBER1 and EBER2 and have been shown binding to several cellular proteins, such as La antigen, EBER-associated protein (now referred to as ribosomal protein L22), RNA-dependent protein kinase and retinoic acid inducible gene 1 [61, 62]. Tsai et al. reported that M81virus exhibited reduced B cell tropism, enhanced epitheliotropism and enhanced viral replication in EBV-infected B cells relative to B95-8 virus. In addition, the spontaneous replication and epitheliotropism could be partly ascribed to polymorphisms within BZLF1 and gp110 proteins of EBV. These findings demonstrate the existence of distinct EBV subtype with enhanced pathogenic potential [47]. The M81-type viruses have distinctive EBER variations (EB-8m) which prevalent in endemic NPC. Thus, whether these variations contribute to the enhanced pathogenic potential of these specific EBV through influencing the functional and immunological potential of EBERs is valuable to be investigated, which could be helpful to clarify the association of EBER variants and NPC, and provide important insights to the roles of EBV in the pathogenesis of NPC.
